# *Histoplasma capsulatum* antigen detection tests as an essential diagnostic tool for patients with advanced HIV disease in low and middle income countries: A systematic review of diagnostic accuracy studies

**DOI:** 10.1371/journal.pntd.0006802

**Published:** 2018-10-19

**Authors:** Mathieu Nacher, Denis Blanchet, Felix Bongomin, Arunaloke Chakrabarti, Pierre Couppié, Magalie Demar, David W. Denning, Félix Djossou, Loïc Epelboin, Nelesh Govender, Terezinha Leitão, Sigrid Mac Donald, Christine Mandengue, Silvia Helena Marques da Silva, Rita Oladele, Maria Mercedes Panizo, Alessandro Pasqualotto, Ruth Ramos, Subramanian Swaminathan, Juan Luis Rodriguez-Tudela, Stephen Vreden, Rosely Zancopé-Oliveira, Antoine Adenis

**Affiliations:** 1 CIC INSERM 1424, Centre Hospitalier Andrée Rosemon, Cayenne, French Guiana; 2 Laboratoire de parasitologie mycologie, Centre Hospitalier Andrée Rosemon, Cayenne, French Guiana; 3 Global Fund for Fungal Infections (GAFFI), Geneva, Switzerland; 4 Postgraduate Institute of Medical Education & Research, Chandigarh, India; 5 Service de Dermatologie Vénéréologie, Centre Hospitalier Andrée Rosemon, Cayenne, French Guiana; 6 Service des maladies infectieuses et tropicales, Centre Hospitalier Andrée Rosemon, Cayenne, French Guiana; 7 Centre for Opportunistic, Tropical and Hospital Infections, National Institute for Communicable Diseases (NICD), Johannesburg, South Africa; 8 Universidade Federal do Ceará, Fortaleza, Brazil; 9 SRCS, Academic hospital Paramaribo, Paramaribo, Suriname; 10 Université des Montagnes, Bangangté, Cameroon; 11 Evandro Chagas Institute, Belem, Brazil; 12 College of Medicine University of Lagos, Lagos, Nigeria; 13 Instituto Nacional de Higiene "Rafael Rangel", Caracas, Venezuela; 14 Universidade Federal de Ciências da Saúde de Porto Alegre, Porto Alegre, Brazil; 15 National Care and Treatment Center, Georgetown, Guyana; 16 Global Hospitals, Chennai, India; 17 Fundação Oswaldo Cruz, Rio de Janeiro, Brazil; Universidad de Antioquia, COLOMBIA

## Abstract

**Introduction:**

Disseminated histoplasmosis, a disease that often resembles and is mistaken for tuberculosis, is a major cause of death in patients with advanced HIV disease. Histoplasma antigen detection tests are an important addition to the diagnostic arsenal for patients with advanced HIV disease and should be considered for inclusion on the World Health Organization Essential Diagnostics List.

**Objective:**

Our objective was to systematically review the literature to evaluate the diagnostic accuracy of *Histoplasma* antigen tests in the context of advanced HIV disease, with a focus on low- and middle-income countries.

**Methods:**

A systematic review of the published literature extracted data on comparator groups, type of histoplasmosis, HIV status, performance results, patient numbers, whether patients were consecutively enrolled or if the study used biobank samples. PubMed, Scopus, Lilacs and Scielo databases were searched for published articles between 1981 and 2018. There was no language restriction.

**Results:**

Of 1327 screened abstracts we included a total of 16 studies in humans for further analysis. Most studies included used a heterogeneousgroup of patients, often without HIV or mixing HIV and non HIV patients, with disseminated or non-disseminated forms of histoplasmosis. Six studies did not systematically use mycologically confirmed cases as a gold standard but compared antigen detection tests against another antigen detection test. Patient numbers were generally small (19–65) in individual studies and, in most (7/10), no confidence intervals were given. The post test probability of a positive or negative test were good suggesting that this non invasive diagnostic tool would be very useful for HIV care givers at the level of reference hospitals or hospitals with the infrastructure to perform ELISA tests. The first results evaluating point of care antigen detection tests using a lateral flow assay were promising with high sensitivity and specificity.

**Conclusions:**

Antigen detection tests are promising tools to improve detection of and ultimately reduce the burden of histoplasmosis mortality in patients with advanced HIV disease.

## Author summary

Disseminated histoplasmosis, a disease that often resembles and is mistaken for tuberculosis, is a major cause of death in patients with advanced HIV disease. Histoplasma antigen detection tests are an important addition to the diagnostic arsenal for patients with advanced HIV disease and should be considered for inclusion on the World Health Organization Essential Diagnostics List. Our objective was to systematically review the literature to evaluate the diagnostic accuracy of Histoplasma antigen tests in the context of advanced HIV disease, with a focus on low- and middle-income countries. Systematic review of the published literature extracted data on comparator groups, type of histoplasmosis, HIV status, performance results, patient numbers, whether patients were consecutively enrolled or if the study used biobank samples. At the end of the screening process we included a total of 16 studies in humans for further analysis. Most studies included used a heterogeneous group of patients, often without HIV or mixing HIV and non HIV patients, with disseminated or non-disseminated forms of histoplasmosis. Patient numbers were generally small in individual studies and, in most of these, no confidence intervals were given. When considering the diagnostic accuracy of *Histoplasma* antigen detection tests evaluated among consecutive HIV-infected patients with confirmed histoplasmosis, the performance of the tests was good. These non invasive diagnostic tools would be very useful for HIV care givers at the level of reference hospitals or hospitals with the infrastructure to perform ELISA tests. Antigen detection tests are promising tools to improve detection of and ultimately reduce the burden of histoplasmosis mortality in patients with advanced HIV disease.

## Introduction

Histoplasmosis was first described in Panama in 1906 in a patient who appeared to have miliary tuberculosis, a confusion that is still very much present today.[1] Disseminated histoplasmosis has been an AIDS-defining infection since 1987.[2,3] Recent estimates using different methods converge on the conclusion that, in Latin America, each year, over 22000 HIV-infected patients get disseminated histoplasmosis and that between 5000 and 10000 HIV-infected persons die from it, mostly for lack of diagnosis.[4] A multicentre study in Latin America reported a 79% increase in the hazard of dying among culture negative “tuberculosis” cases, the author concluded that the patients had another diagnosis, presumably a good illustration of the confusion that is so frequent between tuberculosis and histoplasmosis.[5] In French Guiana, among consecutive HIV patients hospitalized for infectious symptoms (fever, isolated or with other symptoms), 42% of those with CD4<200 had culture-confirmed histoplasmosis, and 85% of those with CD4<50 had histoplasmosis[6]; In Fortaleza, of 378 consecutively admitted HIV patients, 164 (43%) had microscopically confirmed histoplasmosis[7]; In Panama, 7.65% of patients with an HIV infection had culture-positive *H*. *capsulatum*[8], in Guatemala, and in Colombia histoplamosis and TB are the main opportunistic infections;[9,10] In Venezuela, in patients with AIDS, histoplasmosis was documented in 29 of 66 (44%) autopsies performed[11,12]. Most histoplasmin prevalence studies took place before the AIDS epidemic was identified, because histoplasmin testing is no longer performed. They however are a good marker for the current endemicity of *Histoplasma* in the countries where they were performed.[13] In all the above regions, these past studies showed that approximately 30% of the population was reactive to a histoplasmin skin test, thereby illustrating the ubiquity of the fungus[14]. These few studies illustrate the importance of histoplasmosis among HIV-infected patients in Latin America, however much of the continent has no data and often no diagnostic capacity.[4,15,16] But the data gap is even worse in other parts of the world. Cases have been reported in many parts of Africa.[17] In Cameroun a prospective study found that 13% had microscopically confirmed histoplasmosis[18], preliminary unpublished results in South Africa using antigen detection showed that 10–15% of hospitalized HIV-infected patients with a CD4 count <100 had positive antigenemia. In South Asia, South East Asia, China histoplasmosis has also been increasingly reported in HIV patients[19,20]. Overall, there are enormous data gaps regarding the global burden of histoplasmosis, and the lack of simple diagnostic tools creates a vicious circle where the absence of data perpetuates the low awareness of the importance of the problem and the lack of research on the topic.[21] Diagnostic tests are thus crucial to improve public health and to improve the care of patients with advanced HIV disease who are most at risk of developing disseminated histoplasmosis. Cohort studies have shown that incidence increases rapidly as CD4 counts fall below 200 cells per mm3.[22] The isolation of the pathogen may be performed using direct examination of tissue biopsy (identification of yeast-like forms in tissue from bone marrow, lymph nodes, liver, intestine and other organs…), allowing strong suspicion of the diagnosis ultimately confirmed by culture, which often takes over a month and requires expertise and a BSL2/3 laboratory because of the hazard of inhaling the fungal microconidia.[23] Treatment should be presumptive (Amphotericin B or itraconazole depending on the severity) given the delays of fungal culture, and waiting for results to start treatment may delay care for weeks and lead to the patient’s death. [24,25] The invasive procedures, the mycological expertise required and the important delays have led to search for alternative diagnostic methods using non invasive, diagnostic tests giving rapid results to the physician. *Histoplasma* antigen detection tests have been used in the USA for over 30 years[26], but rarely in tropical regions[27–29]. Antigen detection tests are the simplest diagnostic method that could be implemented in low and middle income countries in order to diagnose HIV patients with advanced disease and/or severe illness including a TB-like presentation.

There have been 2 systematic reviews on antigen detection tests.[30,31] One included a meta-analysis but with some methodological issues. First, this metaanalysis included a study on antibody detection with the antigen detection studies. Secondly, the meta-analysis pooled sensitivity and specificity of different *Histoplasma* antigen detection tests, which is debatable. Overall, both systematic reviews concluded that antigen detection tests in general were sensitive and specific diagnostic tools. However, the publication of new studies and concerns for some study limitations and the variation they introduce led us to review the literature in the specific context of advanced HIV disease, with a special focus on low and middle income countries. The systematic review aimed at providing evidence-based arguments for the essentiality of *Histoplasma* antigen detection test in persons with advanced HIV in low and middle income countries.

## Methods

### Ethics statement

This analysis did not deal with individual patient data but with published data, which does not require regulatory approval.

A systematic review of published articles and conference abstracts evaluating *Histoplasma* antigen detection was conducted. PubMed, Scopus, Lilacs and Scielo databases were searched for published articles in English, Spanish and Portuguese between 1981 and 2018. The search terms usedwere: “histoplasmosis” and “antigen detection”, or “histoplasmosis” and “antigenuria”, or “histoplasmosis” and “antigenaemia”. Articles reporting studies in humans and the diagnostic accuracy of antigen detection tests were retained. To ensure completeness, we crosschecked with 2 recent systematic reviews to determine whether we had not missed important studies[30,31]. Finally we looked at Food and Drug administration reports of the Immuno-Mycologics (IMMY) Alpha study.[32] Then, because we were evaluating rigorous studies in HIV patients with disseminated histoplasmosis, articles with culture as a gold standard comparator, and including consecutive severely ill HIV-infected patients with disseminated histoplasmosis were retained. This decision was based on our focus on patients with advanced HIV disease and our assumption that among these patients disseminated histoplasmosis is a major killer. Thus, ideally, all patients (cases and controls) should be HIV-infected and all histoplasmosis patients should be microbiologically confirmed histoplasmosis (gold standard), because from a clinician’s point of view this would reflect the diagnostic challenge when facing a patients with unspecific signs of infection and advanced HIV disease. The studies retained used different diagnostic tests therefore we decided not to synthesize their results in a meta-analysis. Supporting information files show the PRISMA checklist and the PRISMA flow diagram.

From the reported sensitivities and specificities we computed pre and posttest odds, positive and negative likelihood ratios, and posttest probabilities for different hypothesized proportions of histoplasmosis prevalence and for both positive and negative test results. The posttest odds was the multiplication of the pretest odds (hypothesized prevalence) by the positive likelihood ratio; the posttest probability was posttest odds/(1+posttest odds). Pretest and posttest odds, positive and negative likelihood ratios, and posttest probabilities were calculated for scenarios of *Histoplasma* prevalence of 1%, 10%, 20%, and 40% in patients with advanced HIV disease. When missing in the published article, we calculated 95% confidence intervals. We computed the point estimates but also the lowest and highest values derived from the 95% confidence interval. Data were analyzed using STATA 13 (STATA Corporation, college station, Texas).

## Results

Table 1 shows the 16 studies retained and their characteristics. Studies on *Histoplasma* antigen detection tests included heterogeneous groups of patients, some (n = 2) without HIV or others (n = 3) mixing HIV and non HIV patients [32–34], with disseminated or non-disseminated forms of histoplasmosis, which may introduce great variation in measuring the diagnostic accuracy of a test. In 4 studies it was not clear whether patients were HIV-infected or not (Table 1). The control groups, when present, were also different: to calculate specificity some studies used non fungal controls, while others did not, thus potentially introducing variability. Four studies did not use mycologically confirmed cases as a gold standard but compared antigen detection tests against another antigen detection test, calculating sensitivity and specificity in the absence of a gold standard[35–38], instead of looking for agreement using split samples. Patient numbers were generally small (usually ranging from 19 to 65 histoplasmosis cases, the largest compiling 158 cases) and, in most individual studies, no confidence intervals were given. Finally, many studies used stored samples and failed to include consecutive patients. The first study in AIDS patients by Wheat *et al*. performed a comparison with a gold standard of 61 AIDS cases with microscopically proven disseminated histoplasmosis and 30 AIDS controls (total obtained by adding controls with different opportunistic infections). The assay had a very high sensitivity (96.7% (95% CI = 88.6–99.6)) and specificity (100% (95% CI = 88.4–100)).[26,39] Receiver operator characteristic (ROC) curves or likelihood ratios were not calculated in the paper. When calculating the post-test probabilities with the lower bounds of the 95% confidence intervals for sensitivity and specificity, a worst case scenario, the posterior probability of a positive test was 5% if prevalence was 1%, 38% for a 10% prevalence, 58% for a 20% prevalence and 78% for a 40% prevalence. The calculations using 96.7% and 100% point estimates yielded a 98.9% posterior probability for prevalence at 1%. The patients, however, were apparently not included consecutively. The second generation test from MiraVista was apparently tested using consecutive AIDS patients with disseminated histoplasmosis and 100 controls without fungal infections, who did not have HIV or AIDS[40]. We thus excluded the study from our analysis. Regarding the IMMY Alpha, although the samples (in data submitted to the FDA) were tested using microscopically confirmed histoplasmosis as a reference standard[32], it is not clear what proportion of patients were HIV-positive, or what proportion were disseminated histoplasmosis. The study was thus not further analyzed.

**Table 1:** Details on study design of studies evaluating *Histoplasma* antigen detection in Humans with disseminated histoplasmosis.

Study	Year	Test	HIV	Consecutive patients	Territory	Gold standard	Regulatory approval	number of histoplasmosis cases	number of controls	confidence intervals
Wheat et al.[26]	1986	RIA	no	no	USA	yes	no	88	nocontrols	no
Wheat et al.[39]	1989	RIA	yes	no	USA	culture	no	61	27	no
Zimmerman et al.[41]	1989	AP-ELISA HRP-Elisa/RIA	no	no	USA	Culture + clinical suspicion	no	19	24 systemic fungal infections and 17 "others"	no
Gomez et al.[33]	1997		mix	no	Colombia	culture	no	35 serum 16 urine	48 serum 20 urine	no
Conolly et al.[40]	2007	MV2^nd^gen ELISA	yes	no	USA	culture?	no	65	100	no
FDA[32]	2008	IMMY alpha	mix?	no	USA	culture/pathology	yes	47	231	yes
Cloud et al.[35]	2007	IMMY alpha/mv	?		USA	no	yes	?	?	
Le Monte et al.[36]	2007	IMMY alpha/mv	?	no	USA	no	no/yes	?	?	
Gutierrez et al.[42]	2008	MV	yes	yes?	USA/Panama	clinical suspicion aids	no	21 Panama/65 USA	0 no controls	
Scheel et al.[28]	2009	CDC EIA	yes	yes	Guatemala	culture	no	41	197	yes
Hage et al.[34]	2011	MV	mixed	yes?	USA multicentric		no	158 disseminated	229	no
Theel et al.	2013	MV/IMMY ASR galactomannan	?	no	USA	no	no	?	?	
Caceres et al.[27]	2014	CDC EIA	yes	yes	Colombia	culture	no	28	174	yes
Theel et al.	2015	MV/IMMY ASR galactomannan	?	yes	USA	no	no	?	?	no
Caceres et al.[29]	2018	IMMY ASR galactomannan	yes	yes	Colombia/Guatemala	culture	in process	63	526	yes
Caceres et al.[43]	2018	MV LFA	yes	?	Colombia/Guatemala	culture	no	19serum	28 serum from non- HIV controls (bacterial/fungal infections & healthy)	no

**Fig 1:**
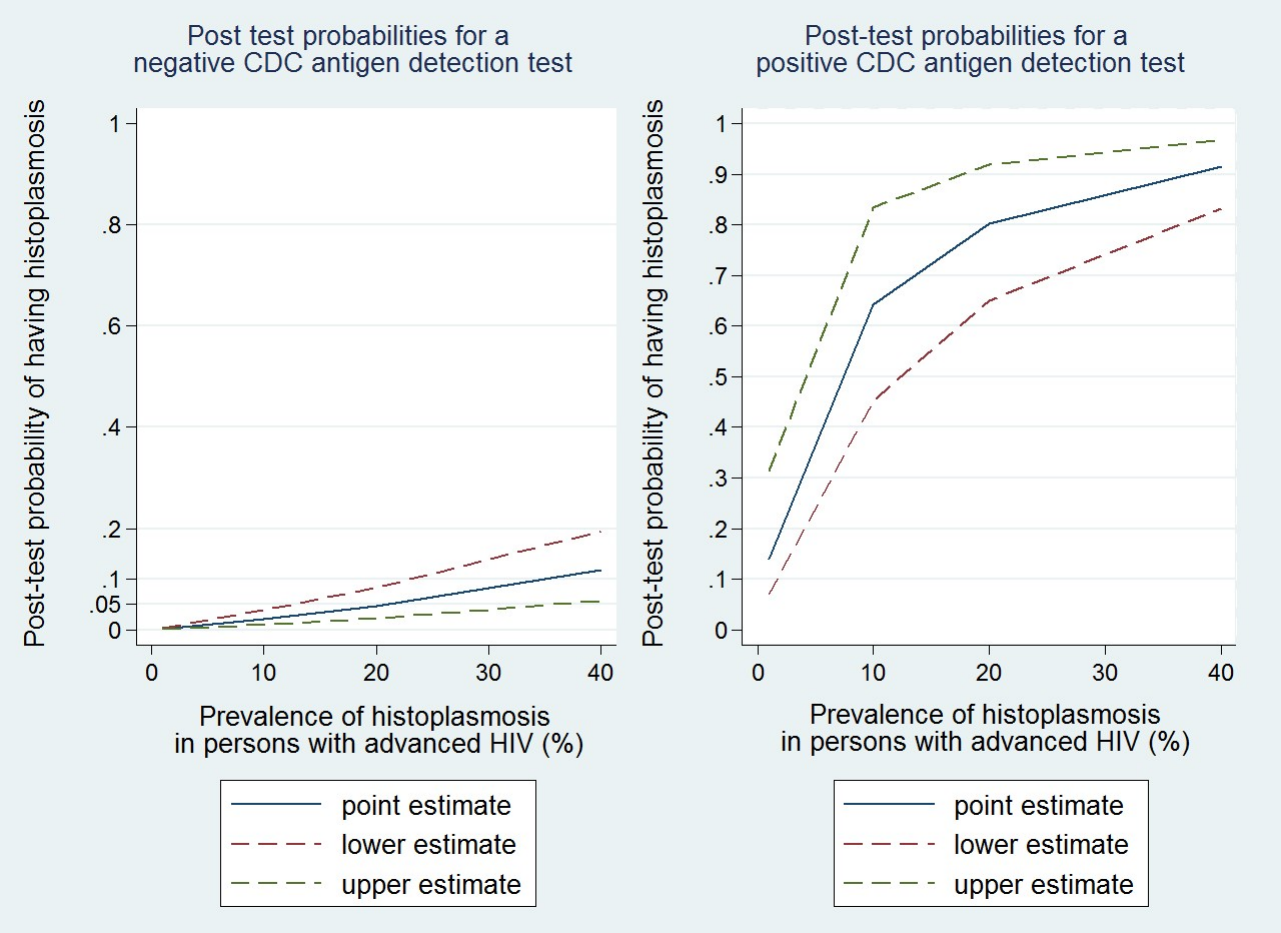
Pre and post test probabilities for the CDC polyclonal *Histoplasma* antigen detection test.

**Fig 2:**
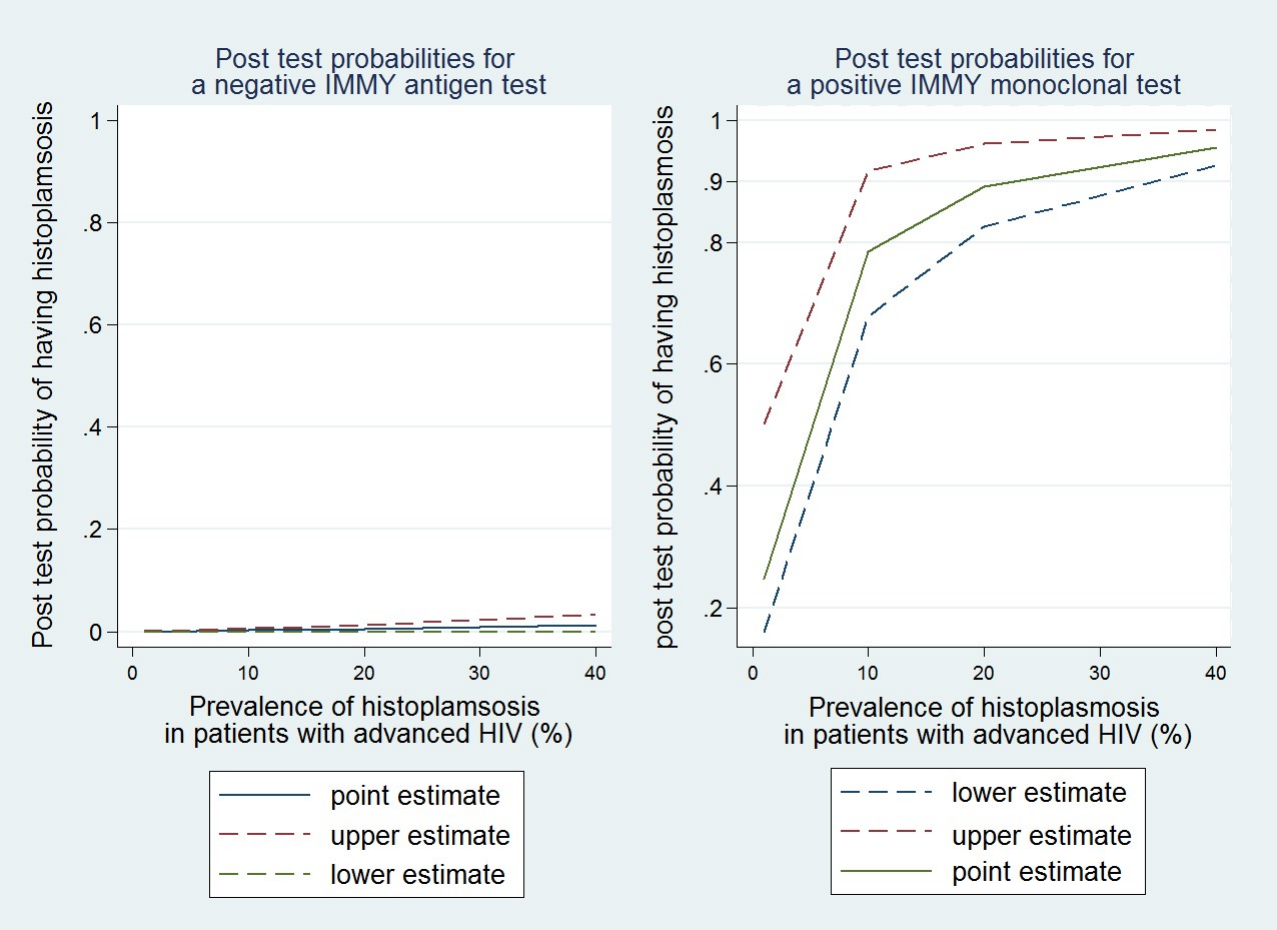
Pre and post test probabilities for the IMMY monoclonal *Histoplasma* antigen detection test.

After retaining studies which consecutively enrolled HIV-seropositive patients with a comparison against the gold standard of culture only 3 studies remained. These studies compared 2 different antigen tests (the CDC polyclonal antigen test and the Immy monoclonal antigen test) to culture, all 3 studies having taken place in 2 populations in Latin America.[27–29] The CDC test in urine had a sensitivity of 81% (95%CI = 67–91%) and a specificity of 95%(95%CI = 91–98%). The area under the ROC curve was 0.87 (95% CI, 0.80 to 0.95), and positive and negative likelihood ratios were 16.1 (7.4–45.5)) and 0.2 (0.09–0.36), respectively. Thus for areas of low prevalence, there was a low post test probability when the CDC test was positive, but the post test probability increased rapidly with prevalence (Fig 1). On the contrary in areas with high prevalence a negative CDC test was still associated with a 0.11 probability of having the disease. Overall the IMMY monoclonal test performed better than the CDC test in a similar Latin American context (Fig 2).

For the IMMY monoclonal antigen detection when using the manufacturer instructions test sensitivity was 98% (95%CI = 95–100%) and specificity was 97% (95%CI = 96–99%). The areas under the ROC curve were 0.99 for the quantitative determination and 0.97 for a semi quantitative adaptation. The results in the patients from Guatemala and from the patients in Colombia showed similar performances. The positive likelihood ratiowas 32.6 (95% CI = 19–100) and the negative likelihood ratio was 0.02 (95%CI = 0–0.05).

Finally, an abstract presented at the 2018 ISHAM conference compared a new lateral flow assay from MiraVista diagnostics used on culture confirmed HIV-associated histoplasmosis and non-HIV infected controls. The point estimates for this new point of care test were 95% sensitivity and 82% specificity.

## Discussion

The first antigen detection test was developed in the USA in 1986, using polyclonal antibodies against *Histoplasma* galactomannan.[26] It was initially a radio immune assay and was subsequently modified as an EIA.[34,40] This test has very good reported sensitivity and specificity but it is only performed in Indianapolis at MiraVista Diagnostics and it is not FDA approved. In the context of low and middle income countries this test is thus apparently not a viable option.[25] In 2007, a polyclonal FDA approved EIA was commercialized (IMMY Alpha).There were conflicting results after comparisons with the MiraVista test with a variety of methodological issues ranging from the types of patients selected in the study, their HIV status, the test procedure, and other biases. If we focus on item 18 of the recent PRISMA DTA guidelines, it is noteworthy that there was a conflict of interest (many of the authors pointing the IMMY alpha’s lack of sensitivity were linked to MiraVista a company with a large share of the United States’ market of histoplamosis diagnosis).[44] Despite these methodological issues, the early controversy has stood in the way of a widespread use of the IMMY alpha.[32,35,36] The IMMY test has been recently modified using monoclonal antibodies which have greatly improved its sensitivity.[29] Given the lack of diagnostic test for low and middle income countries the CDC’s mycotic diseases branch developed a polyclonal EIA that was evaluated in consecutive HIV patients in Colombia and Guatemala, and was successfully implemented in Brazil, Suriname, and French Guiana.[28] Although there is an agreement that antigen detection was a very sensitive and specific non-invasive test,[23] the published studies have greatly varied in design and in comparison groups. The burden of histoplasmosis in patients with advanced HIV-disease has been greatly underestimated,[4,20] in this specific context looking at consecutive HIV-infected patients with advanced disease, the antigen tests seem to have great value. The CDC test and the MiraVista EIA are not likely to be submitted for FDA approval, and the IMMY monoclonal EIA is apparently in the process of FDA approval for commercialization. There is currently 1 test with FDA approval: IMMY alpha.[32] We did not retain the analysis from the FDA data because it was not clear that all cases only had disseminated histoplasmosis or a mix of clinical forms, and if any or some cases had HIV.

Beyond diagnostic accuracy, the epidemiological context is important to keep in mind when interpreting the results of a test. We do not precisely know the burden of histoplasmosis in HIV-positive patients in many parts of the world. Until now, antigen detection tests have been used successfully for research in a number of tropical countries including, Colombia, Guatemala, Panama, Brazil, French Guiana, and Suriname. This suggests that antigen detection is appropriate for different regional clades.[27–29,42] Costing will be an important aspect since diagnosis is mostly a problem in low and middle income countries with high HIV prevalence.[25] Although it has been argued that doing the antigen test on urine and serum increased sensitivity,[45] recent studies have shown there was no significant benefit to repeat the test.[46] In resource limited countries, this would unnecessarily increase costs. There are still discussions on whether urine is more sensitive than serum, this should be further evaluated using a proper study design in consecutive patients with advanced HIV disease. The test is an EIA format and therefore requires a minimum infrastructure with an EIA reader, electricity, refrigeration, and organization with sample transport, conservation, batching EIA runs … Therefore, reference hospitals and large hospitals may be equipped to perform such a test. The cost of an EIA for low and middle income countries is not available yet. However, costs often increase dramatically with multilayered distribution networks that are necessary for a manufacturer in a high income country to reach the end users in countries where they have no presence. In this situation, each additional intermediary level will amplify costs, whatever the manufacturers’ initial cost. The fact that reference laboratories are targeted would possibly make it easier for manufacturers to streamline distribution and avoid unnecessary cost hikes. When point of care antigen detection format becomes available this would allow further scale up of diagnosis to the most remote health care facilities. The recent communication by Caceres *et al*. on the miravista lateral flow assay seems very promising and could radically change things if the test confirms its diagnostic performance and if it becomes available in all endemic countries at an affordable price.

The WHO has published an essential diagnostics list[47]. We believe that *Histoplasma* antigen detection tests should be included because this would greatly raise awareness of clinicians and public health authorities, a crucial first step to reduce unnecessary AIDS deaths for lack of a proper diagnosis.[4] While the performance measurements of the test are a first step, the most important question would be will these tests make a difference in terms of saving lives? Thus, as recommended by WHO, a PICO (Population, Intervention, Comparison, Outcome) question regarding antigen detection tests could be: Among patients with advanced HIV-disease, are *Histoplasma* antigen detection tests better than the present standard of diagnosis to improve the diagnosis of histoplasmosis and reduce mortality? Since the most frequent standard of diagnosis is no diagnosis for histoplasmosis, the answer to this question may seem straightforward: it is better to make a diagnosis than to miss a diagnosis. In terms of outcome, for diagnosis and mortality, data from Colombia showed that the availability of antigen testsand training dramatically increased the number of diagnoses.[48] In French Guiana, increased awareness and diagnostic progress led to very important increases in the number of diagnoses and a 4 fold reduction of case fatality at one month.[49] Increased awareness alone will have huge benefits on the number of patients diagnosed and treated, an antigen detection test may even show more of the hidden part of the Histoplasmosis “iceberg”.

In conclusion, the studies on *Histoplasma* antigen detection methods have suffered from great heterogeneity, partly because it is challenging to get sufficient numbers of consecutive HIV-infected culture proven disseminated histoplasmosis cases. Excellence centers in low and middle income countries seem better positioned to perform these studies. As shown by the pioneering Colombian and Guatemalan collaboration studies, the evaluation of future antigen test upgrades (lateral flow assays) should rest on well-designed studies in consecutive patients with HIV and confirmed histoplasmosis test that can best be achieved through a North-South collaboration. Furthermore, 30 years after the first test, *Histoplasma* antigen detection tests manufacturers should go through the regulatory approval process in order to make tests available in low and middle income countries which have been an underappreciated potential market. Meanwhile, the available antigen detection tests, should be included in the essential diagnostics list to start mapping the global burden of disseminated histoplasmosis. This would greatly accelerate the goal of having diagnostic tests and effective drugs for disseminated histoplasmosis in most hospitals in endemic countries be achievable.[50]

## Supporting information

S1 PRISMA Checklist (DOCX)

S1 PRISMA Flow Chart (DOCX)
