# Supplementary material for: Histoplasma capsulatum antigen detection tests as an essential diagnostic tool for patients with advanced HIV disease in low and middle income countries: A systematic review of diagnostic accuracy studies
Source: PLoS Negl Trop Dis. 2018 Oct 19;12(10):e0006802. doi: 10.1371/journal.pntd.0006802 (PMC6209380; doi:10.1371/journal.pntd.0006802)
Supplement: S1 PRISMA Flow Chart — (DOCX) [file pntd.0006802.s002.docx]

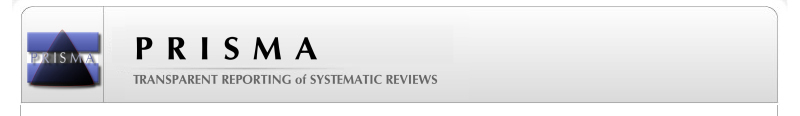
**S1 PRISMA Flow Chart**

Full-text articles excluded:

-No gold Standard (n=6)

Non-HIV or mixed HIV/non-HIV patients
(n =4)

-Non consecutive HIV patients (n=3)

Studies included in quantitative synthesis (n =3)

(meta-analysis not done)

Studies included in qualitative synthesis
(n =16)

Full-text articles assessed for eligibility
(n =16)

Records excluded
(n =1312)

Records screened
(n =1328)

Records after duplicates removed
(n =1328)

Additional records identified through other sources
(n =2)

## Identification

## Eligibility

## Included

## Screening

Records identified through database searching
(n =1326)
